# Gender specific survival rates after deceased donor liver transplantation: A retrospective cohort

**DOI:** 10.1016/j.amsu.2022.103933

**Published:** 2022-06-05

**Authors:** Uri Gabbay, Assaf Issachar, Michal Cohen-Naftaly, Marius Brown, Eviatar Nesher

**Affiliations:** a Department of Epidemiology and Preventive Medicine, School of Public Health, Sackler Faculty of Medicine, Tel-Aviv University, Tel Aviv, Israel; b Quality Unit, Rabin Medical Center, Beilinson Hospital, Petach Tikva, Israel; c Department of Internal Medicine, Sackler Faculty of Medicine, Tel-Aviv University, Tel Aviv, Israel; d Liver Institute, Rabin Medical Center, Beilinson Hospital, Petach Tikva, Israel; e Department of Surgery, Sackler Faculty of Medicine, Tel-Aviv University, Tel Aviv, Israel; f Organ Transplantation Ward, Rabin Medical Center, Beilinson Hospital, Petach Tikva, Israel

**Keywords:** Deceased donor, Gender, Liver transplantation (LT), Outcome, sex

## Abstract

**Background:**

According to the literature, there are sex allocation inequalities in liver transplantation (LT). Sex disparities in outcomes after LT have been debated. This study aimed to evaluate sex-specific outcomes after LT, specifically short-term mortality and long-term survival rates.

**Methods:**

A retrospective cohort of the entire LT series from to 2010–2019 in a single center in which the inclusion criteria were adults ≥18 YO age who underwent primary deceased donor LT. Mortality rate was evaluated within 30 days and 6 months. Survival rate was evaluated at 1,3 and 5 years of age.

**Results:**

A total of 240 primary and deceased donor LTs (153 men and 87 women) were included. Mean age 55.2Y men and 51.6Y women (p = 0.02). Hepatocellular carcinoma (HCC) was the direct indication in 32.7% of the men and only 17.4% of the women. The leading primary liver morbidities were viral hepatitis (B, C, and D) in 38.3% (N = 92) and nonalcoholic steatohepatitis (NASH) in 20.8% (N = 50) of patients. Thirty-day mortality was 14%, which was significantly higher in men (18%) than in women (8%). Survival rates after 5 years were 64.9% and 78.3%, respectively. Multivariate analysis through logistic regression that included age, direct indication, MELD, and primary liver morbidity revealed statistically significant female to male Odds-Ratio of 0.4 in 30 days, 6 m mortality and a statistically significant higher long-term survival.

**Conclusions:**

Our observations revealed better female outcomes, namely, lower short-term mortality and higher long-term survival. Given the consistency after stratification and given the multivariate analysis, this is unlikely to be attributable to confounders. Such findings suggesting consistently better female outcomes have not been previously reported; hence, multi center study is encouraged.

## Highlights

• Gender outcome disparities after liver transplantation (LT) is debated.
• We present retrospective cohort of 240 consecutive LT in a single center.
• We found significant lower short-term mortality rates and better long-term survival in women.
• The outcome disparity was consistent in most underlying liver diseases.
• We had not identified factors that may confound sex outcome disparity.

## Abbreviation

AIH: Autoimmune hepatitis
ELTR: Europe Liver Transplantation Registry
ESLD: End-stage liver diseases
FHF: Fulminant hepatic failure
HBV: Viral hepatitis B
HCC: Hepatic cell carcinoma
HCV: iral hepatitis C
LT: Liver transplantation
MELD: Model for End-Stage Liver Disease
NASH: Nonalcoholic steatohepatitis
OLD: Other liver diseases
PBC: Primary biliary cholangitis
PSC: Primary sclerosing cholangitis
SRTR: Scientific Registry of Transplant Recipients

## 1 Introduction

Despite significant advancements in the treatment of liver diseases, liver transplantation (LT) remains a significant treatment option (sometimes the only alternative) for patients with end-stage liver disease (ESLD), acute fulminant liver failure, or hepatocellular carcinoma [1]. However; the availability of organ donors is limited.

In the last few decades, there has been major improvement in post-LT survival. The implementation of the Model for End-stage Liver Disease (MELD) score for prioritizing allocating organs for patients on the waiting list improved accessibility for LT, as well as waiting list mortality [2,3]. The implementation of the MELD scoring system has also successfully addressed a significant portion of racial disparities, but remains controversial regarding gender disparities in LT [4,5].

The data on sex differences mostly pertain to waiting list mortality and accessibility for LT. Possible confounders for sex disparity may include age, direct indication for LT, primary liver disease, and its severity. Previous researches also suggested differences in creatinine and body size, and the higher odds for women to became “too sick” for transplantation [[6], [7], [8]].

Whether sex also affects post-transplant survival remains controversial. In a study from Heidelberg, Germany, female sex was associated with higher 90-day mortality in high MELD (>20) but not in low MELD (<20) [9]. In the U.S. Scientific Registry of Transplant Recipients (SRTR) study, women had greater odds of receiving a low-quality graft than men, but there was no difference in graft survival [10]. Europe Liver Transplantation Registry (ELTR) study showed a statistically significant higher 10-year survival rate in women (66% vs. 59%, *P* < 0.0001) [11]. Another German study showed better long-term survival (up to 20 years) in women undergoing LT [12]. There were also sex differences in primary liver disease and indications for LT, as well as comorbidities that may affect post-LT mortality risk [13].

Our center is a tertiary adult care hospital that has pioneered the Israel national transplantations program and is still a considerable performer in the diversity of transplantations (i.e., heart, lung, kidney, and liver transplantations). On an average, 35 liver transplantations (deceased and live donors) were performed annually.

The aim of this study was to compare sex-specific outcomes after primary deceased donor LTs, namely short-term mortality and long-term survival.

## 2 Materials and methods

Retrospective cohort of the entire series of consecutive transplantations at a single liver transplantation center, between 2010 and 2019. Inclusion criteria were patients ≥18 YO (adults), who underwent primary, deceased donor LT. Excluded were re-transplantations and multi-organ transplantations (e.g., liver kidney). Entry was at liver transplantation day. The end of follow-up was either death or survival at the end of the study, on 30th June 30, 2020. Mortality rate was evaluated within 30 days and 6 months. Survival rate was evaluated at 1,3 and 5 years of age.

The primary endpoint of our study was sex-specific post LT survival. Secondary targets were sex-specific survival stratified by age group, direct indication for LT, MELD, and primary liver morbidity.

The study protocol was approved by the institutional review board (Helsinki Committee), which waived the need for written informed consent.

The study was registered in the Research Registry, UIN: researchregistry7515 (https://www.researchregistry.com/browse-the-registry#home/registrationdetails/61d7555849b193001ef9e366/).

### 2.1 Definitions and outcomes

The duration of follow-up for patients who died (during follow-up) was the period between the LT and death date. Accordingly, the duration of follow-up of patient who survived until the end of follow-up was calculated as the difference between the LT date and June 30, 2020 (end of the study follow-up).

### 2.2 Data collection

The retrieved data included demographics, anthropometrics, basic clinical characteristics, direct indication for LT, primary liver disease, clinical features of liver disease such as ascites and hepatic encephalopathy, MELD (registration for LT and prior to LT), laboratory data, date of transplantation, and outcome.

### 2.3 Stratification

Age was stratified into three groups:18-49Y, 50-64Y, and ≥65 years. Direct indication for transplantation was defined as either malignancy (HCC) or ESLD alone (HCC excluded). The MELD score was divided into two groups: low (<20) and high (≥20). Six primary liver disease groups were defined: viral hepatitis (HBV + HCV), nonalcoholic steatohepatitis (NASH), autoimmune hepatitis (AIH), cholangitis-related diseases ((primary sclerosing cholangitis (PSC) + primary biliary cholangitis (PBC)), fulminant hepatic failure (FHF), and other liver diseases (that were not specifically mentioned) (OLD).

### 2.4 Analysis

We evaluated short-term outcome as 30d mortality, and 6 m mortality. The long-term outcomes were 1,3 and 5 years survival rate. We calculated post LT gender specific survival for the entire series by direct indication for LT (with and without HCC), MELD severity, age group, and primary liver disease.

Statistical analyses were performed using SPSS version 25 (IBM Ltd., US, 2018). Continuous variables are presented as mean ± SD and evaluated by Student's t-test and analysis of variance (ANOVA). Frequencies were analyzed using Fisher's exact test and chi-square test for the comparison of discrete variables. Multivariate analysis was performed using a logistic regression analysis. Multivariate survival was analyzed using Kaplan-Meier and Cox regression analyses to compare long-term survival. Differences were considered statistically significant when the P ≤ 0.05. The work has been reported in line with the STROCSS criteria [14].

## 3 Results

During 2010–2019, 261 LTs from deceased donors were performed at our center. Excluded were 21, which left after exclusions 240 primary, deceased donor LT.

### 3.1 Patient baseline characteristics

The baseline patient characteristics are summarized in Table 1. The mean age was slightly and statistically significantly younger in women than men by 3.5 years. Mean height and weight were significantly lower in women as expected, nevertheless, BMI was nearly identical by sex.

**Table 1:** Overall and gender specific baseline characteristics at LT.

Characteristic	All (240)	Men (153)	Women (87)	*P*-value
Mean age (years)	53.9	55.2	51.6	0.02
Mean MELD score at listing	17.7	17.0	19.1	0.03
Mean MELD score at listing when primary indication for LT is ESLD	19.5	19.0	20.3	0.20
Mean MELD score at listing when primary indication for LT is HCC	13.2	12.9	14.0	0.54
Mean MELD score on transplantation	19.8	18.8	21.5	0.02
Mean MELD score on transplantation when primary indication for LT is ESLD	22.3	21.4	23.5	0.09
Mean waiting time from listing (days)	344	327	374	0.56
Mean Height (centimeters)	169	174	160	<0.001
Mean Weight (kilogram)	78.8	83.3	70.7	<0.001
Mean BMI (kg/m^2^)	27.7	27.6	28.0	0.7
Mean Creatinine	1.2	1.0	1.6	0.36
Mean INR	1.7	1.6	1.9	0.01
Mean Bilirubin	5.6	4.7	7.3	0.005
Donor age (years)	52.5	53.2	51.2	0.45
Donor age over 50YO	59%	59.5%	57.5%	0.76
Hospital stay (days)	19.5	17.1	23.9	0.09
Average follow up (days)	1359	1234	1581	0.02
Time until death (those who die) (days)	98	111	75	0.5
Primary liver disease (N) in percentages				
Viral hepatitis (all types) (92)	38.3	41.8	32.2	0.17
NASH (50)	20.8	21.6	19.5	
Autoimmune hepatitis (13)	5.4	3.3	9.2	
Fulminant hepatic failure (7)	2.9	3.9	1.1	
PSC + PBC (35)	14.6	13.1	17.2	
All other (43)	17.9	16.3	20.7	
HCC as primary indication for LT (65)	27.2	32.7	17.4	0.007
Ascites % (167)	69.6	70.6	67.8	0.38
HRS % (17)	7.1	6.5	8.0	0.42
Esophageal varices % (117)	48.8	52.3	42.5	0.09
Hepatic encephalopathy % (105)	43.8	45.8	40.2	0.24
Cholangitis % (12)	5.0	3.9	6.9	0.24
TIPS % (5)	2.1	2.6	1.1	0.40

The mean MELD at listing was 17.7, at listing and 19.8 at LT, which was significantly higher in women than in men. There was a longer waiting time for LT in women, but the difference was not statistically significant.

The fraction of HCCs as direct indications for LT was significantly lower (nearly half) in women than in men. MELD was not significantly different by sex when HCC was the direct indication, but was significantly different by sex when HCC excluded. The distribution of primary liver disease was not identical between sexes, but was not statistically significant. There were no significant differences in clinical disease complication rates between women and men. There were no statistically significant differences in donor age or rate of donor above 50 years of age.

### 3.2 Outcome by gender

**Fig. 1:**
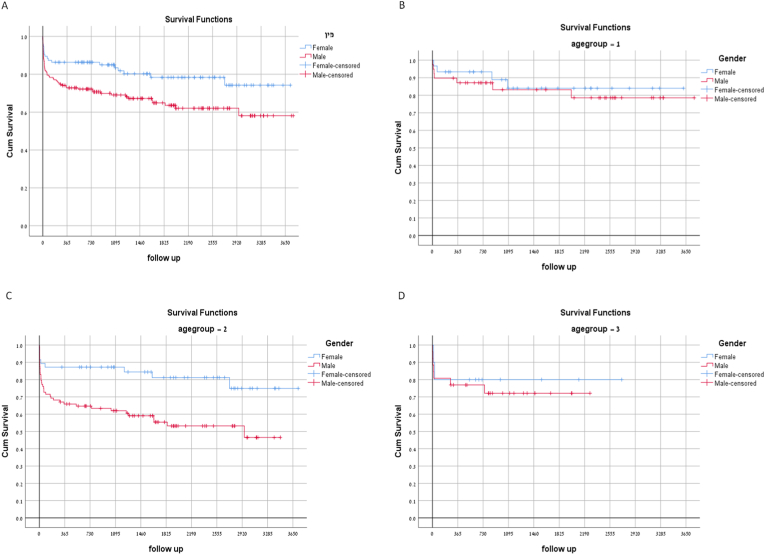
Gender specific survival curves (A), and by age group:(B) 18-49y, (C) 50-65y, and (D) 65+.

Sex-specific outcomes are presented in Table 2 and Fig. 1A. Statistically significant differences were observed in the 30-day mortality (8% in women vs. 17.6% in men, *P* = 0.03) and 6-month mortality (11.5% vs. 23.5%, *P* = 0.02). The survival curve presented in Fig. 1A revealed better female survival. 1-, 3-, and 5- year survival rate (83.3%, 83.4%, and 78.3% in women vs. 72.8%, 69%, and 64.9% in men respectively, *P* = 0.004).

**Table 2:** Gender specific outcome by primary indication for LT.

	All indications				HCC as primary indication for LT				Solely ESLD as primary indication for LT (HCC excluded)			
	All (240) (%)	Males (153) (%)	Women (87) (%)	*P*-value	All (65) (%)	Males (50) (%)	Women (15) (%)	*P*-value	All (175) (%)	Males (103) (%)	Women (72) (%)	*P*-value
30-day mortality	14.2	17.6	8.0	0.03	9.2	10	6.7	0.58	16.1	21.4	8.5	0.017
6-month mortality	19.2	23.5	11.5	0.02	12.3	12.0	13.3	0.60	21.8	29.1	11.3	0.004
1-year survival	77.7	72.8	86.3	0.004	87.7	88.0	86.7	0.61	74.2	66.2	86.1	0.002
3-year survival	74.3	69.0	83.4		82.7	83.7	78.8		71.3	62.4	84.2	
5-year survival	69.8	64.9	78.3		76.4	75.5	78.8		66.4	60.6	77.8

### 3.3 Outcome by direct indication for LT

The outcomes of direct indications for LT are presented in Table 2.

HCC; Gender-specific survival curves were similar and not significantly different between sexes when HCC was the direct indication for LT.

HCC excluded; Gender-specific survival curves were considerably and statistically significantly better in women when the direct indication for LT was solely ESLD.

### 3.4 Outcome by MELD at registration

The outcomes of MELD at registration are presented in Table 3. When solely ESLD was the direct indication for LT (HCC excluded), 5 year survival rates were 52.4% in men and 78.1% in women when MELD was <20 (p = 0.014) and 64.1% in men and 87.1% in women when MELD was ≥20 (p = 0.05), both of which in favor of woman outcome.

**Table 3:** Gender specific outcome by MELD category on registration.

Direct indication for LT		MELD<20				MELD≥20			
	Outcome measure	All (%)	Males (%)	Women (%)	p-value	All (%)	Males (%)	Women (%)	p-value
HCC as direct indication	30-day mortality	13.2	15.7	8.8	0.9	16.0	21.6	6.7	0.5
	6-month mortality	17.6	19.6	14.0	0.4	22.2	31.4	6.7	0.34
	1-year survival	80.5	77.5	86.0	0.6	75.1	64.6	93.3	0.91
	3-year survival	76.6	71.3	86.0		71.7	64.6	83.9	
	5-year survival	70.3	62.4	80.3		71.7	60.3	83.9	
Solely ESLD (HCC excluded)	30-day mortality	16.3	21.3	9.3	0.1	15.7	21.4	7.1	0.1
	6-month mortality	22.1	27.9	14.0	0.09	21.4	31.0	7.1	0.02
	1-year survival	75.0	67.2	86.0	0.014	75.4	64.1	92.9	0.05
	3-year survival	70.9	59.9	86.0		73.1	64.1	87.1	
	5-year survival	65.4	52.4	78.1		73.1	64.1	87.1	

### 3.5 Outcome by age groups

The outcomes by age group are presented in Fig. 1B,C, and D. Short- and long-term outcomes were better in women than in men, but not statistically significant in the 18-49Y group (Fig. 1B) and the 65+ YO group (Fig. 1D). For the 50–65 YO group (Fig. 1C), the 6-month mortality rate was considerably and statistically significantly lower in women than in men (12.8% vs. 30.7%, respectively, P = 0.016). As shown in Fig. 1C, survival rates in the 50-65Y group after 1, 3, and 5 years were significantly higher for women than for men (88%, 83%, and 79% vs. 64%, 60%, and 55%, respectively, P = 0.005).

### 3.6 Outcome by primary liver disease

Outcome by gender and by primary liver disease classification is presented in Table 4.

**Table 4:** Overall and gender specific outcome by primary liver disease.

		Both genders	Males	Women	
**NASH**	**N**	50	33	17	*P*-value
	30 days mortality	10.0%	12.1%	5.9%	0.44
	6 month mortality	16.0%	18.2%	11.8%	0.44
	One year survival	79%	73%	85%	0.30
	Three years survival	70%	65%	85%	
	Five years survival	66%	60%	75%	
**Autoimmune hepatitis**	**N**	13	5	8	
	30 days mortality	0%	0%	0%	–
	6 month mortality	15.4%	40%	0%	0.13
	One year survival	79%	33%	93%	0.058
	Three years survival	70%	50%	87%	
	Five years survival	66%	35%	75%	
**PSC** + **PBC**	**N**	35	20	15	
	30 days mortality	17.1%	20.0%	13.3%	0.48
	6 month mortality	22.9%	25.0%	20.0%	0.53
	One year survival	84%	79%	81%	0.83
	Three years survival	67%	79%	81%	
	Five years survival	64%	60%	63%	
**Viral hepatitis (all types)**	**N**	92	64	28	
	30 days mortality	18.5%	23.4%	7.1%	0.05
	6 month mortality	23.9%	29.7%	10.7%	0.04
	One year survival	74%	80%	84%	0.043
	Three years survival	70%	74%	83%	
	Five years survival	66%	51%	82%	
**Fulminant hepatic failure**	**N**	7	6	1	
	30 days mortality	0%	0%	0%	–
	6 month mortality	0%	0%	0%	–
	One year survival	100%	100%	100%	–
	Three years survival	100%	100%	100%	
	Five years survival	100%	100%	100%	
**All Other ESLD**	**N**	43	25	18	
	30 days mortality	14.0%	16.0%	11.1%	0.50
	6 month mortality	14.0%	16.0%	11.1%	0.50
	One year survival	81%	78%	85%	0.25
	Three years survival	81%	74%	85%	
	Five years survival	81%	74%	85%	

Viral hepatitis patients had significantly lower mortality rates after 30d and 6 m in women than in men (7.1% vs. 23.4%, *P* = 0.05, and 10.7% vs. 29.7%, *P* = 0.04, respectively). Women had significantly better survival than men after 1, 3, and 5 years (84%, 83%, and 82% vs. 80%, 74%, and 51%, *P* = 0.043, respectively).

NASH and OLD patients had considerably better survival in women than in men, but the differences were not statistically significant.

Patients with AIH had no documented mortality within 30d in both sex. Women had considerably lower mortality rates after 6 m but the difference was not statistically significant. There were considerably better survival rates in women after 1,3 and 5 years, but borderline statistically significant (P = 0.058).

Patients with cholangitis-related diseases patients (PSC + PBC) of both sexes had very similar survival rates.

#### 3.6.1 Multivariate analysis

The results of multivariate logistic regression analysis for short-term mortality (30 days and 6 m) are presented in Table 5. The results revealed that age and sex were statistically significant variables (p < 0.05), but neither HCC nor MELD, (at transplantation) or primary liver disease was a primary indication.

**Table 5:** Multivariate analysis for short and long term outcome post LT.

30 days mortality logistic regression model															
Variable	OR				OR 95% CI								P value		
**Age**	1.040				1.002				,		1.079		0.044		
**Female gender**	0.377				0.152				,		0.934		0.035		
**HCC as primary indication**	0.379				0.131				,		1.098		0.074		
**MELD at transplantation**	1.012				0.962				,		1.065		0.65		
**Primary liver disease**	1.168				0.926				,		1.476		0.19		
**6 months mortality logistic regression model**															
**Variable**			OR			OR 95% CI								P value	
**Age**			1.038			1.005				,		1.074		0.028	
**Female gender**			0.388			0.177				,		0.850		0.018	
**HCC as primary indication**			0.40			0.156				,		1.019		0.055	
**MELD at transplantation**			1.017			0.972				,		1.064		0.47	
**Primary liver disease**			1.038			0.846				,		1.272		0.72	
**Multivariate Cox-regression 5 years survival analysis**															
**Variable**		OR		OR 95% CI											P value
**Age**		1.028		1.004			,	1.053							0.023
**Female gender**		0.417		0.232			,	0.748							0.003
**HCC as primary indication**		0.596		0.313			,	1.133							0.114
**MELD at transplantation**		1.012		0.979			,	1.046							0.49
**Primary liver disease**		0.998		0.865			,	1.152							0.98

Variable(s) entered on step 1: age, Gender, HCC as primary indication, MELD at transplantation, primary liver disease.

#### 3.6.2 Long-term survival

The Cox regression for 5 years survival multivariate analysis is presented in Table 5. The results revealed that age and female sex were statistically significant variables (p < 0.05), but neither HCC was a primary indication nor MELD score at transplantation or primary liver disease.

## 4 Discussion

Our study pointed towards better women survival after deceased donor liver transplantation. Whenever considerable sex-specific survival differences are detected, potential confounders should be excluded. A better outcome was evident when the direct indication for LT was solely ESLD (HCC excluded). When the direct indication for LT was HCC, survival was similar for both sexes. Women's better outcomes were evident independent of the MELD category (above or below 20). Better women outcomes were considerable and statistically significant for the 50-65Y group. A better outcome was evident in most primary liver disease groups (excluding combined cholangitis PSC + PBC, in which sex-specific survival was similar). However, a statistically significant difference was noted only when the underlying liver disease was viral hepatitis.

Women were slightly younger on average, which is a positive prognostic factor, but other female characteristics were negative prognostic factors such as higher average MELDs both at registration and LT, longer waiting period before LT, and lower fraction of HCC as a direct indication for LT (HCC is associated with better prognosis).

Multivariate analysis of short- and long-term outcomes (including age, direct indication for LT, Meld, and primary liver morbidity) revealed that sex (female) is an independent protective prognostic factor for survival. The question of whether sex affects post-transplant survival has been controversial in previous studies. Our results differ from those of studies that showed that female sex is associated with worse outcomes for patients with hepatitis C undergoing LT [15,16].

Recent studies support our findings. A study from Heidelberg, Germany, Bruns et-al. Showed that being female was a positive predictor of postoperative 90-day and 1-year mortality in the MELD>20 group [9]. In a recent study from the European Liver Transplant Registry (ELTR), Germani et al. showed that in a group of 46,334 L T patients, women had a significantly better survival rate up to 10 years after LT [11]. Schoening et-al demonstrated a better survival rate in women for up to 20 years after LT (*P* = 0.017) in 313 patients [12].

There are several possible factors that have associated post LT outcomes differently by sex. In a U.S. Scientific Registry of Transplant Recipients (SRTR) study, Mathur et al. showed that in a large group of 19,249 liver transplant recipients, women had greater odds of receiving a low-quality graft than men; however, there was no difference in graft survival [10]. Menopause is associated with higher rates of weight gain and increases in central fat mass, both risk factors for developing NASH and metabolic syndrome [17]. However, several studies have shown that male sex is a risk factor for new-onset diabetes [18,19] and post LT obesity [20]. men have also a higher long-term risk of post LT cardiovascular disease [21,22]. In recent years, many studies have demonstrated the influence of sarcopenia on mortality before and after LT. Male sex is an independent predictor of sarcopenia [23], and low muscle mass was also associated with worse post LT survival in men but not in women [24]. In a study from the US SRTR, Bhat et al.. Showed that the male sex is an independent predictor of post LT de novo malignancy [25]. Finally, male sex was associated with poorer survival in patients aged >65 YO of age undergoing LT, but not in women [26]. Estrogen can also be involved in better outcomes for women, as demonstrated in a study showing that E2/ERa signaling increase in bilirubin metabolism might contribute to better post-LDLT surgery outcomes and hepatocyte function recovery during the liver regeneration process [27].

Our study has several limitations, including its modest volume and single-center series over several years. Nevertheless, the indication for LT and the percentage of these indications are in concordance with other LT centers in Europe and the US. Our center is a prominent referral center for LT in Israel. Moreover, the allocation of deceased donor organs to the patient and transplantation center is determined independently by the Israeli National Center of Transplantation.

We became aware of the apparently sex outcome disparities following quality assurance outcome evaluation. We had review the literature and found debated findings. We are aware that we present a modest single center experience. However, there is also an advantage as confounding effect can be eliminated in regard with staff (same staff), protocols (same protocols), organ preparation (identical organ's preparation), surgical technique (same surgical technique) or post operative care (same post operative care). The advantages of a single center also include similar pre-transplantation care, same proceedings, and the same liver institute that evaluate and follow transplanted patients in the long term.

We are not claiming our findings represent universal phenomenon but we wish to share our findings to the scientific and professional community to raise their concern and encourage discussion whether a multicenter evaluation is justified.

The consistencies of the findings by stratification and through multivariate analysis strongly decrease concern that the differences in outcome between genders were due to random effect (“chance”). Better female outcomes were consistent even when statistical significance was not reached, which also strengthened the likelihood of phenomenon validity. The modest group size may have prevented statistical significance.

The study was a retrospective cohort based of existing database. We had not identified differences that may confound gender disparities in both short and long term outcomes. However our retrospective cohort was limited to the available documented data.

## 5 Conclusions

We demonstrated a better female outcome after liver transplantation in the short and long term. Given the consistency of the results by the underlying liver disease and the multivariate analysis, this is unlikely to be attributable to known and available confounders. Consistently better female outcomes have not been previously reported. We are not claiming this is universal phenomenon but we wish to share our findings with the scientific and professional community to raise their concern. We suggest that a multicenter evaluation targeting outcome sex disparities should be considered.

## Ethical approval

Helsinki Committee, Rabin Medical Centre No:649-2021-RMC, November 21, 2021.

## Source of funding

None.

## Author contribution

**Uri Gabbay** - had initiated and designed the study, performed the data analysis, interpreted the findings and co-draft the manuscript. **Assaf Issachar** - had design the study, review the literature, contributed to the data capture, interpreted the findings and co-draft the manuscript. **Michal Cohen-Naftaly** - had reviewed the literature, interpreted the findings, review and revised the manuscript. **Marius Brown** - had reviewed the literature, evaluated the data capture, interpreted the findings, review and revised the manuscript. **Eviatar Nesher** had initiated and designed the study, contributed to the data capture, interpreted the findings, reviewed the literature, review and revised the manuscript. All authors confirm the final manuscript.

## Trial registry number

1. Name of the registry: Research Registry
2. UIN: researchregistry7515
3. https://www.researchregistry.com/browse-the-registry#home/?view_2_search=7515&view_2_page=1

## Guarantor

Uri Gabbay.

## Data availability statement

Data is not available publicly.

## Provenance and peer review

Not commissioned, externally peer-reviewed.

## Declaration of competing interest

None.
